# Sound-Induced Intracellular Ca^2+^ Dynamics in the Adult Hearing Cochlea

**DOI:** 10.1371/journal.pone.0167850

**Published:** 2016-12-13

**Authors:** Dylan K. Chan, Stephanie L. Rouse

**Affiliations:** Department of Otolaryngology-Head and Neck Surgery, University of California, San Francisco, United States of America; University of South Florida, UNITED STATES

## Abstract

Ca^2+^ signaling has been implicated in the initial pathophysiologic mechanisms underlying the cochlea's response to acoustic overstimulation. Intracellular Ca^2+^ signaling (ICS) waves, which occur in glia and retinal cells in response to injury to activate cell regulatory pathways, have been proposed as an early event in cochlear injury. Disruption of ICS activity is thought to underlie Connexin 26-associated hearing loss, the most common genetic form of deafness, and downstream sequelae of ICS wave activity, such as MAP kinase pathway activation, have been implicated in noise-induced hearing loss. However, ICS waves have only been observed in neonatal cochlear cultures and are thought to be quiescent after the onset of hearing. In this study, we employ an acute explant model of an adult, hearing cochlea that retains many *in vivo* physiologic features to investigate Ca^2+^ changes in response to sound. We find that both slow monotonic changes in intracellular Ca^2+^ concentration as well as discrete ICS waves occur with acoustic overstimulation. The ICS waves share many intrinsic features with their better-described neonatal counterparts, including ATP and gap-junction dependence, and propagation velocity and distance. This identification of ICS wave activity in the adult, hearing cochlea thus confirms and characterizes an important early detection mechanism for cochlear trauma and provides a target for interventions for noise-induced and Connexin 26-associated hearing loss.

## Introduction

Acoustic trauma is among the most common causes of permanent sensorineural hearing loss [1]. Though many physiologic sequelae of cochlear trauma have been identified, including activation of reactive oxygen species and hair-cell apoptosis, the initial pathophysiologic effects of acoustic overstimulation are poorly understood. In particular, experimental limitations have largely precluded evaluation of cellular-level processes in a sound-stimulated, mature-hearing cochlea.

In adult guinea pigs, noise exposure is associated with an acute increase in endolymphatic ATP levels [2], raising the possibility that ATP release into scala media is an early event in noise-induced ototoxicity. This finding was bolstered by a series of studies in neonatal organotypic mouse cochlear cultures implicating intracellular Ca^2+^ signaling (ICS) waves in this process. In response to mechanical trauma, hair-cell ablation, or exogenous ATP exposure, ATP is released through connexin-based hemichannels on the apical surface of supporting cells, activating apical purinergic receptors, intracellular Ca^2+^ second messengers, and release of Ca^2+^ from endoplasmic reticular stores [3–5]. This intracellular Ca^2+^ signal then propagates across supporting cells in a regenerative ICS wave that is dependent upon intercellular gap junctions [6,7]. Cells through which these ICS waves pass subsequently show upregulation of ERK and JNK [3,8], two mitogen-activated protein kinase (MAPK) pathway effectors that have been implicated in hair-cell survival [9] and apoptosis [8,10]. These *in vitro* studies in neonatal cochlear explants thus suggest a link between acoustic trauma, endolymphatic ATP, ICS activity, and downstream MAPK effectors, and are further supported by *in vivo* findings of noise-induced phospho- and total- MAPK modulation in the cochlea [11]. Finally, by analogy to similar spontaneous and trauma-induced ICS wave activity in the central nervous system and retina [12,13], this paradigm has been proposed as an early mechanism for detection of cochlear injury.

ICS wave activity has also been indirectly implicated in the pathogenesis of hearing loss associated with Connexin 26 (Cx26) dysfunction, which is the most widespread cause of genetic hearing loss worldwide [14]. Cx26-based gap junctions are primarily thought to be critical for K^+^ circulation through supporting-cell networks in the cochlea, but have also been shown to play many other distinct roles in cochlear development, hair-cell electromotility, and cochlear homeostasis [15–17]. An additional major functional role of Cx26 may be to support ICS wave activity. Humans with the V84L mutation in Cx26, which has intact junctional conductance but absent transmission of IP_3_, a Ca^2+^ second messenger necessary for ICS activity, have hearing loss [18], suggesting that ICS activity is protective and its disruption central to the pathogenesis of Cx26-associated hearing loss. Finally, a recent study showed that Cx26 conditional knockout mice have increased susceptibility to noise-induced hearing loss, suggesting a common pathophysiologic mechanism underlying both forms of deafness [19].

These studies implicate ICS waves in the pathogenesis of both noise-induced and Cx26-associated hearing loss, the most common forms of acquired and genetic hearing loss in humans, respectively. The ICS wave phenomenon, however, has only been directly observed in neonatal cochlear cultures prior to the onset of hearing and is thought to become quiescent following development of mature hearing, by postnatal day 16 (P16) [20]. Though purinergic signaling has been shown to evoke intracellular Ca^2+^ transients in individual supporting cells using a hemicochlea preparation of the adult mouse cochlea [21], no direct evidence exists demonstrating that the adult, hearing cochlea retains the capacity to support regenerative ICS waves. Furthermore, it is unknown whether these waves, or other changes in intracellular Ca^2+^ dynamics, can be induced in response to physiologic or pathophysiologic sound stimuli.

In this study, we have used an acutely isolated preparation of the adult gerbil cochlea in which the apical and basolateral surfaces of the sensory epithelium are separated and bathed respectively in artificial endolymph and perilymph, and acoustically-stimulated mechanoelectrical transduction is preserved [22]. This preparation allows us to evaluate early changes in intracellular Ca^2+^ and discrete ICS wave activity in cells of the organ of Corti upon acoustic overstimulation. Demonstration of sound-induced Ca^2+^ changes and ICS wave activity in this *in vitro* explant model of the adult, mature-hearing cochlea may provide insight into the early pathophysiologic mechanisms of noise-induced hearing loss as well the role of Cx26 dysfunction in genetic deafness.

## Methods

### Animal management

This study involved euthanasia by CO2 and cervical dislocation/decapitation of gerbils and mice, and anesthesia with ketamine and xylazine. All animal protocols were approved by the UCSF Institutional Animal Care and Use Committee.

### Organotypic culture of the neonatal mouse cochlea

Organotypic cultures from P3 wild-type C57/Bl6 mice were established [23]. Briefly, P3 mice were euthanized and decapitated. The cochlear duct was isolated, opened, and plated on glass coverslips with Cell-Tak (Corning, Bedford, MA) with the apical surface of the epithelium facing up. Cultures were incubated overnight at 37°C and 5% CO_2_ in DMEM-F12 + 10% FBS and 50 mg/mL ampicillin, and used for experiments after 24 hours in culture.

### *In vitro* preparation of the adult gerbil cochlea

To assess intracellular Ca^2+^ dynamics in supporting cells of the adult mammalian cochlea in response to acoustic stimuli, we used an *in vitro* explant preparation of the middle turn of the gerbil cochlea [22]. Gerbils were used because, among common rodent animal models, their temporal-bone anatomy uniquely allows for minimally traumatic isolation of a single cochlear turn with adequate visualization of the organ of Corti. 8-week-old female Mongolian gerbils were euthanized and the middle cochlear turn excised with the bony labyrinth and modiolus retained. Dissection was performed in dissecting solution (DS; 145 mM NaCl, 3 mM KCl, 0.25 mM CaCl_2_, 0.25 mM MgCl_2_, 2 mM Na-pyruvate, 5 mM D-glucose, 10 mM Na_2_HPO_4_, pH 7.35). The cochlea was mounted using tissue-compatible cyanoacrylate glue (Histoacryl, TissueSeal) on a plastic coverslip with a hole in its center. Windows were made in the bone separating the apical and middle turns, and in the bone separating the middle and basal turns, thus providing a direct visual and acoustic path across the sensory epithelium. Reissner's membrane was removed and the scalae sealed in the apical and basal directions with additional glue. The preparation was mounted in a custom-built two-chamber recording apparatus with the tectorial membrane facing up. The lower compartment (bathing the basilar membrane) was filled with artificial perilymph (AP; 145 mM NaCl, 3 mM KCl, 1.3 mM CaCl_2_, 0.9 mM MgCl_2_, 2 mM Na-pyruvate, 5 mM D-glucose, 10 mM Na_2_HPO_4_, pH 7.35) and the upper compartment (bathing the tectorial membrane) was filled with artificial endolymph (AE; 150 mM KCl, 25 μM CaCl_2_, 2 mM Na-pyruvate, 5 mM D-glucose, 10 mM K_2_HPO_4_, pH 7.35). Specimens were dissected and experiments completed within 60 minutes of euthanasia.

Acoustic stimulation was provided by an earphone (ER-2, Etymotic Research) driven by a power amplifier (Macrotech 2400, Crown) connected to an experimental computer (Optiplex 9020, Dell). Sound was delivered to the basilar membrane via the lower chamber of the two-chamber recording apparatus. A 1 kHz pure-tone stimulus was used, which is near the typical resonant frequency of the preparation [22]. Mechanical movement was measured by video stroboscopic imaging of the inner-hair-cell (IHC) hair bundle. Stroboscopic illumination was provided by a green light-emitting diode (Rebel 530 nm LED, Luxeon Star) driven by 10-V DC power supply (HY5003, MDJA) and gated with a 1:10 on:off square-pulse duty cycle (BuckPuck DC Driver, Luxeon Star) driven by a waveform generator (3312A, Hewlett-Packard). Illumination at ~1001 Hz coincident with 1000 Hz acoustic stimulation frequency yielded stroboscopic illumination at ~1 Hz that was captured with a high-speed CMOS camera (ORCA-Flash4.0, Hamamatsu). Image analysis was conducted by direct measurement of the IHC hair bundle position on successive still images (ExCap, Hamamatsu; Photoshop 13.0, Adobe Systems).

For this explant preparation, geometric gain between movement of the IHC hair bundle and basilar membrane was previously estimated at unity, and the sensitivity of bundle displacement to SPL of the acoustic stimulus measured at 1.8 μm/Pa [24]. Compared to measurements made in the mammalian cochlea *in vivo*, these values are within the reported range, but may represent an underestimate of the actual SPL delivered [25,26]. In response to high-SPL stimuli, the basilar membrane behaves passively, without compressive nonlinearity associated with cochlear amplification. SPL of the acoustic stimulus for this study was thus extrapolated linearly from IHC hair bundle motion using the previously reported sound-pressure and displacement relationship for this preparation. Microphonic potential was recorded from the sound-stimulated adult explant using two pairs of Ag/AgCl electrodes immersed in the upper and lower compartments of the two-chamber recording apparatus, with a second set of Ag/AgCl electrodes driven by a stimulus isolation unit (A395, World Precision Instruments) providing a transepithelial DC potential to mimic the endocochlear potential [22].

### Fluorescence recovery after photobleaching (FRAP)

To qualitatively evaluate gap-junction conductance, we performed a fluorescence recovery after photobleaching (FRAP) assay [23]. Calcein-AM, a gap-junction-permeant vital dye, was loaded into cells by incubation of neonatal organotypic cultures and adult explants with 5 μM calcein-AM in DS + 0.01 w/v Pluronic F-127 and 250 μM sulfinpyrazole to aid dye uptake and prevent sequestration, respectively, at RT for 15 minutes. After washing with DS, specimens were incubated for an additional 10 minutes at 37°C for dye de-esterification.

Samples were imaged with a 60x, 0.80-NA, water-immersion objective on an upright microscope (BX-51, Olympus) outfitted to receive the collimated output of a 405 nm, 50 mW diode laser (Thorlabs) from a fiberoptic cable. This wavelength is shifted from the optimal absorption of calcein, at 480 nm, but has been shown previously to result in reproducible and significant photobleaching in cochlear explants [23]. The input optics were configured such that an 5-μm-diameter aperture-limited image of the fiberoptic laser output is obtained in the specimen plane. After obtaining baseline calcein fluorescence images, a 1-second laser pulse was used to photobleach a 5-μM-diameter spot. Subsequent calcein fluorescence images were taken every 2 seconds for 2 minutes to measure fluorescence recovery. Fluorescence measurements in regions of interest were performed using MetaFluor software (Molecular Devices).

### Ratiometric intracellular Ca^2+^ imaging

To evaluate intracellular Ca^2+^ concentration ([Ca^2+^]_i_), ratiometric imaging was performed using FURA-2-AM, a cell-permeant dye, in neonatal organotypic cultures [4] and adult explants. Cochleae were loaded by incubation with 16 μM FURA-2 in DS + 0.01 w/v Pluronic F-127 and 250 μM sulfinpyrazole for 15 minutes at RT, followed by a 10-minute wash in DS at 37° for de-esterification. Alternating excitation at 340 and 387 nm was performed using a high-speed fluorescence light source, filter wheel, and shutter (Lambda XL, Sutter Instruments), and emission at 510 nm was recorded using a high-speed CMOS camera. The ratio of emission stimulated upon 340- and 387-nm excitation is proportional to [Ca^2+^]_i_, and was measured in regions of interest using MetaFluor. Calibration of [Ca^2+^]_i_ to FURA-2 fluorescence ratio was performed using a set of calibration standard (F6774, Thermofisher) in water; however, because *in situ* calibration could not be performed in the cochlear preparations, we only report relative changes in [Ca^2+^]_i_ within a single experiment, and not absolute [Ca^2+^]_i_ levels.

ATP was applied to specimens either by bath application, or by using a pneumatic PicoPump to deliver a puff of ATP through a pulled glass micropipette with a 10-μM tip. For direct mechanical damage, a glass micropipette was pulled with a 2–3 μm tip, positioned with a micromanipulator at the reticular lamina above an outer hair cell (OHC), and the back of the pipette holder manually tapped to elicit mechanical trauma. Carbenoxolone (CBX), a general inhibitor of connexin-based channels, apyrase, an ATP-degradation enzyme, gentamicin, an aminoglycoside blocker of hair-cell mechanoelectrical transduction, and thapsigargin, an inhibitor of sarco/endoplasmic reticulum Ca^2+^-ATPase (SERCA), were bath applied.

Each measurement of sound-induced [Ca^2+^] changes in the presence of different drugs was performed in a unique freshly dissected cochlear explant, in order to avoid irreversible effects of acoustic overstimulation. For all measurements of sound-induced [Ca^2+^] changes, baseline 340/387 ratio was measured in the measurement medium (either artificial endolymph or artificial endolymph + the drug indicated) for 2 minutes prior to sound exposure. Sound was then applied for 300 s and 340/387 ratio continuously measured at 2-second intervals. 340/387 ratio values at -60 s, 0 s, +80 s and +300 s relative to sound initiation were calculated as the average of 5 timepoints immediately preceding each mark. The change in 340/387 ratio was then calculated as the difference between the -60-s and 0-s values (for baseline value), or the difference between the +80s or +300s value and the 0-s value (for 80s or 300s values, respectively), and reported as a velocity of change (change in 340/387 ratio per minute) to account for the different measurement intervals. The 80s (early) and 300s (late) values were then corrected for the baseline drift.

### Inducible conditional Cx26 knockout mice

Timed knockout (KO) of Cx26 was accomplished to yield mice with normal hearing at P21 for noise exposure experiments [16,17]. Cx26^loxP/loxP^ mice (E00245, European Mutant Mouse Archive) were crossed with ROSA26^Cre/Esr1^ mice (004847, Jackson Lab) to yield experimental mice in which tamoxifen (TMX) delivery induces expression of Cre recombinase driven by the *Gt(ROSA)26Sor* promoter, excision of exon 2 of *GJB2*, and knockdown of Cx26. Mice underwent intraperitoneal injection of 0.5 mg/10 g body weight 4-hydroxytamoxifen (H7904, Sigma-Aldrich) for three consecutive days from P8-P10.

### Acoustic overstimulation and hearing testing

We examined the effect of acoustic overstimulation in wild-type (WT) and Cx26 conditional KO mice *in vivo*. Hearing was tested *in vivo* by measuring auditory brainstem response (ABR) thresholds in response to broadband clicks in sound field using a standard commercial system (RZ6, Tucker-Davis Technologies) in a soundproof chamber. Hearing was tested at baseline at P21, and noise exposures performed by P25. Animals were exposed to 106-dB octave-band (8–16 kHz) white noise for 60 minutes in a custom-built, calibrated, reverberant sound chamber. Animals were unanesthetized in order to avoid affects of general anesthesia on sound-induced threshold shifts [27]. Click ABR thresholds were measured again at 1, 3, 7, and 18 days after noise exposure.

### Statistical analysis

Values are presented as means or difference in means, and standard errors of the mean. Statistical significance was determined for paired or unpaired parametric data using a t-test. For difference in means, statistical significance was determined when the 95% CI of the difference in means did not overlap zero, thus rejecting the null hypothesis of no difference. Statistical analysis was performed using MatLab (Mathworks, v.8.4).

## Results

### Sound delivery to the cochlear sensory epithelium *in vitro*

**Fig 1:**
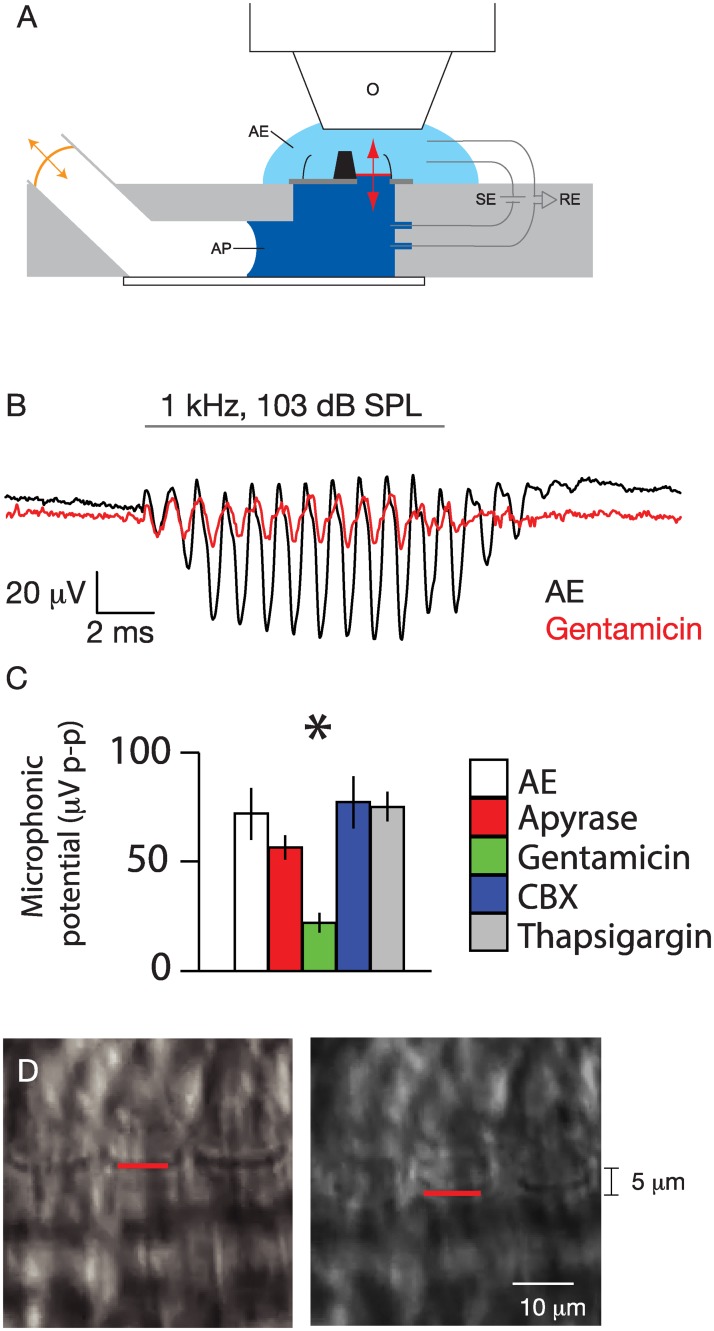
Explant preparation of the adult gerbil middle cochlear turn. A. Schematic diagram of the cochlear explant (black) mounted on a plastic cover slip (dark gray) in a two-chamber recording apparatus (light gray). Delivery of sound pressure with an attached earphone (orange) is transmitted through artificial perilymph (AP, dark blue) to the basilar membrane (red), whose apical surface is immersed in artificial endolymph (AE). Epifluorescence and transmitted-light stroboscopic microscopy are performed with a 60x water-immersion objective (O). Transepithelial potential is provided, and microphonic potential recorded, using paired Ag/AgCl stimulation and receiver electrodes (SE, RE, respectively). B. Microphonic potential. Transepithelial microphonic field potential was recorded in response to 1 kHz, 103 dB SPL sound stimulus (gray line) in artificial endolymph (AE, black) or AE + 1 mM gentamicin (red). C. Effect of drugs on microphonic potential. The microphonic potential was measured in response to 1 kHz, 103 dB SPL sound stimulus in the presence of artificial endolymph (AE, white), or artificial endolymph containing 40 U/l apyrase (red), 1 mM gentamicin (green), 100 μM CBX (blue), or 1 μM thapsigargin (gray). N = 5 explants for each. Bars indicate means, and lines indicate standard errors of the mean; asterisks indicate *p* < 0.05 in unpaired t-test between gentamicin and AE. D. Videostroboscopy of sound-stimulated explant organ of Corti. Stroboscopic still images from an explant preparation undergoing 1 kHz, 103 dB SPL sound stimulation are shown at maximal positive and negative deflection. Radial motion of the IHC hair bundle was used to calibrate the sound-pressure level delivered to the organ of Corti.

A 1-kHz pure-tone sound stimulus was delivered to the apical surface of the adult gerbil cochlear sensory epithelium (Fig 1). Sound-pressure level was estimated by observing the radial excursion of the IHC hair bundle on stroboscopic video. This excursion was measured at 5 μm peak-to-peak at 1 kHz. Based on measurements of geometric gain and sensitivity in this preparation [22,24], this corresponds to 103 dB SPL delivered to the basilar membrane; calibration based upon other published *in vivo* measurements of geometric gain within the organ of Corti (0.7–0.9) [26] and sensitivity of basilar membrane motion to acoustic pressure (0.5–1.8 μm/Pa) [25] yield an estimate of 104–116 dB SPL for the stimulus in air. With a transepithelial potential of +40 mV, a peak-to-peak microphonic potential of 55 μV was recorded in response to a 103 dB SPL, 1-kHz sound stimulus, demonstrating the physiologic viability of the preparation. This response was diminished upon application of 1 mM gentamicin.

### Fluorescence recovery after photobleaching (FRAP)

**Fig 2:**
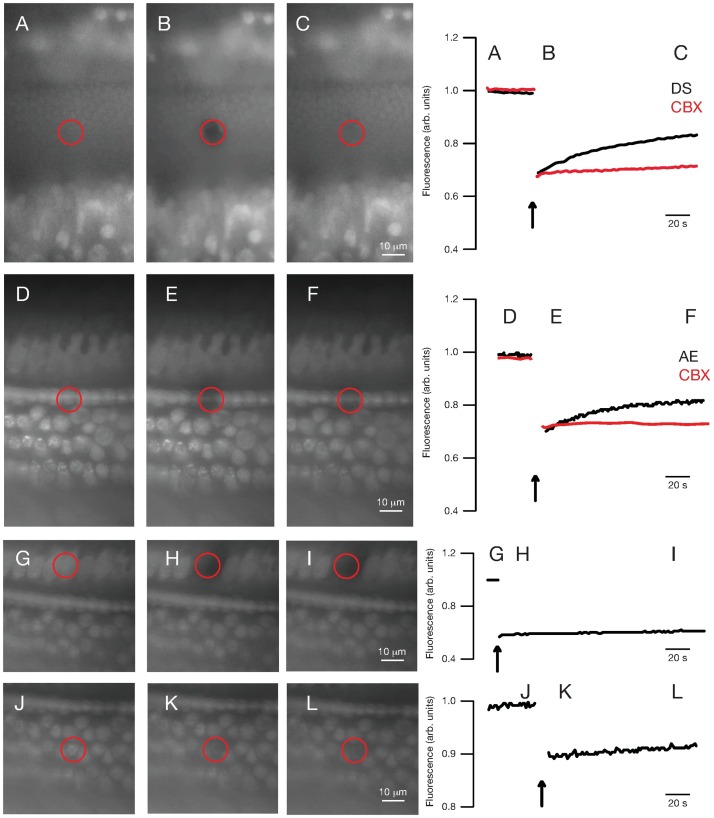
Fluorescence recovery after photobleaching (FRAP). FRAP was performed on supporting cells of the outer sulcus of P3 neonatal cochlear cultures (A-C, top row), as well as on pillar cells (D-F, second row from top), IHCs (G-I, third row from top), and OHCs (J-L, bottom row) from adult cochlear explants. Cells were loaded with calcein dye. After baseline imaging (A, D, G, J), a 5-μm spot was laser-photobleached (B, E, H, K; vertical arrow) and fluorescence recovery measured (C, F, I, L). Fluorescence timecourses were recorded in dissecting solution (DS, for neonatal cultures, black line) or artificial endolymph (AE, for adult explants, black line) or in DS or AE with 100 μM CBX (red lines).

To evaluate gap-junctional connectivity between cells, we assessed recovery of calcein fluorescence after laser photobleaching (Fig 2). After loading with calcein dye and photobleaching a 5-μm-diameter spot with a 405-nm laser, fluorescence recovery was seen in the inner and outer sulcus cells of the neonatal organotypic cochlear culture (Fig 2A–2C). Rate and extent of recovery were qualitatively similar to that seen previously [23]. When assessed in the adult acutely isolated cochlear explant, pillar cells exhibited FRAP (Fig 2D–2F), but not inner (Fig 2G–2I) or outer (Fig 2J–2L) hair cells. FRAP was abolished in both neonatal culture and adult explant preparations upon treatment with 100 μM CBX, a general inhibitor of connexin-based hemichannels and gap junctions (Fig 2A–2F), demonstrating the ability of CBX to attenuate gap-junction conductance in both neonatal culture and adult explant systems.

### Sound-induced intracellular Ca^2+^ dynamics

**Fig 3:**
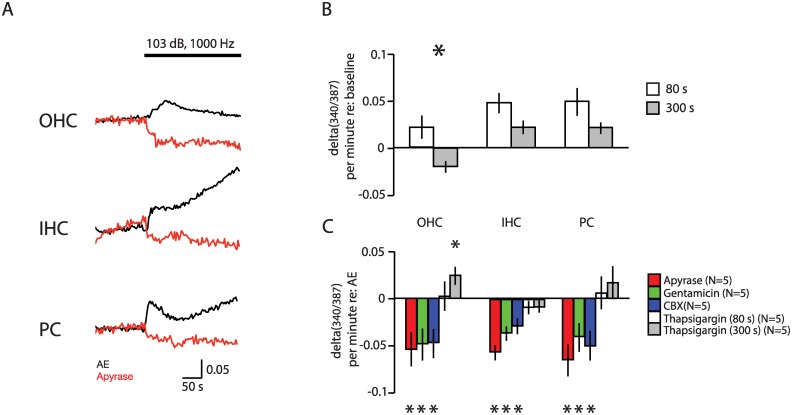
Noise-induced [Ca^2+^]_i_ changes in the gerbil cochlea *in vitro*. A. Sound-induced [Ca^2+^]_i_ changes. Sample traces illustrating [Ca^2+^]_i_ changes in outer hair cells (OHC), inner hair cells (IHC) and pillar cells (PC) in response to application of 1 kHz sound pressure at 103 dB SPL. Black lines: response in artificial endolymph alone. Red lines: response in artificial endolymph containing 40 U/l apyrase. Vertical axis depicts the ratio between fluorescence elicited by 340 nm/387 nm excitation wavelengths, which is proportional to [Ca^2+^]_i_ B. Change in 340/387 ratio_I_ (delta(340/387)) relative to baseline in OHCs, IHCs, and PCs 80 s (white) and 300 s (gray) after the start of a 103 dB SPL, 1000 Hz sound stimulus in artificial endolymph. Bars indicate means and lines indicate standard errors of the mean from 5 independent explants; asterisks indicate *p* < 0.05 in paired t-test between 80s and 300s values. C. Change in 340/387 ratio 80 s after sound initiation was measured in the presence of 40 U/l apyrase (red), 1 mM gentamicin (green), 100 μM CBX (blue), or 1 μM thapsigargin (white). Bars represent means, and lines 95% CI of the difference in delta(340/387) compared to that measured in artificial endolymph (Fig 3b). For thapsigargin, delta(340/387) at 300 s is additionally shown (gray bars), and was compared to the value at 300 s in artificial endolymph alone (N = 5). Asterisks indicate *p* < 0.05 relative to no difference between drug and artificial endolymph.

Stimulation with high levels of sound pressure induced changes in [Ca^2+^]_i_ (Fig 3A). In the presence of artificial endolymph, sound pressure in the form of 103 dB SPL at 1 kHz delivered to the basilar-membrane side of the two-compartment recording chamber induced slow changes in [Ca^2+^]_i_ in OHCs, IHCs, and pillar cells. Preparations were internally consistent in terms of behavior within cell types; that is, in one preparation, all of one cell type behaved similarly. However, preparations varied from one to another. Whereas an early, small increase in [Ca^2+^]_i_ was consistently seen in IHCs and pillar cells (in 5/5 independent preparations), OHCs sometimes exhibited an increase in [Ca^2+^]_i_ (3/5 preparations) and other times showed a decrease in [Ca^2+^]_i_ upon sound stimulation. The rise in [Ca^2+^]_i_ peaked in the first 80 s after initiation of sound, with a subsequent slower phase of increase or, sometimes, reversal back towards baseline; this late Ca^2+^ behavior was highly variable from one preparation to another (Fig 3B).

Previous studies have suggested that this increase in [Ca^2+^]_i_ is due in part to ATP release from connexin-based hemichannels [2–6]. Changes in [Ca^2+^]_i_ were measured in response to 103 dB SPL 1000 Hz sound in the presence of 40 U/l apyrase, to eliminate exogenous ATP stimulation, 100 μM CBX, to inhibit ATP release into the apical compartment through connexin hemichannels, 1 μM thapsigargin, an inhibitor of SERCA, which clears intracellular Ca^2+^ through uptake into endoplasmic reticular stores, and 1 mM gentamicin, a blocker of mechanoelectrical transduction channels (Fig 3C). Upon sound exposure, drugs affected the early Ca^2+^ peak at 80 seconds and longer-term tonic rise differently. Compared with artificial endolymph alone, presence of apyrase, CBX, and gentamicin each decreased Ca^2+^ influx in all three cell types, consistent with dependence of sound-induced Ca^2+^influx upon extracellular ATP, connexin hemichannel function, and mechanoelectrical transduction, respectively. Thapsigargin treatment did not effect initial Ca^2+^ influx, but was associated with increased intracellular [Ca^2+^]_i_ at later timepoints, consistent with its inhibition of cytosolic Ca^2+^ clearance.

Taken together, these results are consistent with the hypothesis that exogenous ATP, connexin-based hemichannel function, and mechanoelectrical transduction are involved in early sound-induced Ca^2+^ influx in the hearing cochlea, whereas SERCA-mediated Ca^2+^ reuptake plays a role in longer-term Ca^2+^ homeostasis.

### Intracellular Ca^2+^ dynamics in neonatal and adult cochleae

**Fig 4:**
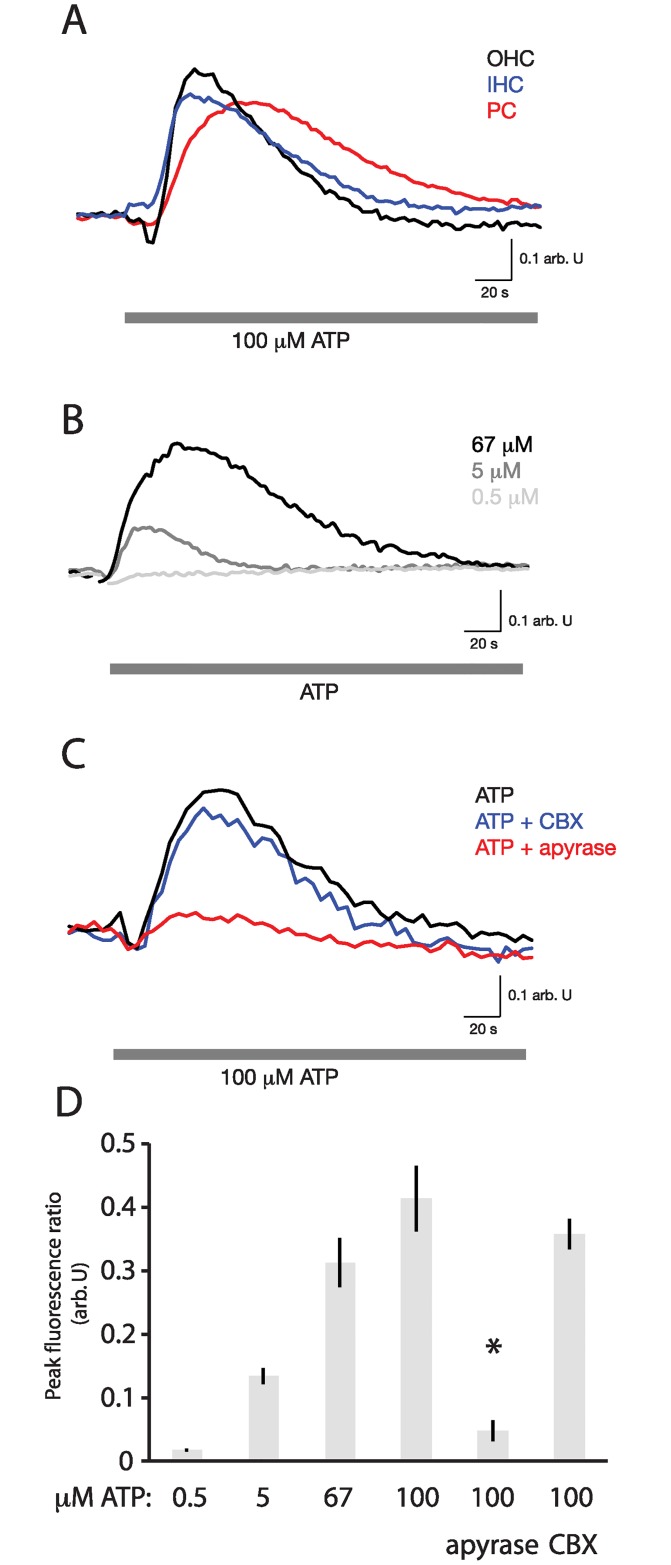
ATP-induced [Ca^2+^]_i_ changes in the gerbil cochlea *in vitro*. A. ATP-induced Ca^2+^ influx into cochlear cells. Bath application of 100 μM ATP in artificial endolymph (gray bar) to a single cochlear explant induces a large increase in fluorescence ratio (340/387) (proportional to [Ca^2+^]_i_) in OHCs, IHCs, and pillar cells (PC). B. Dose-response of Ca^2+^ influx to repeated applications of three different concentrations of ATP in a single explant. C. Pharmacologic response. Ca^2+^ increase in response to 100 μM ATP in a single explant is sensitive to 40 U/l apyrase, but not to 100 μM CBX. D. Peak fluorescence ratio upon treatment with different concentrations of ATP, or upon co-treatment with 40 U/l apyrase or 100 μM CBX, is shown in aggregate (N = 3 explants for each measurement). Asterisk indicates significant attenuation (*p* < 0.01) of the response to 100 μM ATP in the presence of apyrase relative to exposure to 100 μM ATP alone. Bars indicate means; lines indicate standard errors of the mean.

Ratiometric Ca^2+^ imaging was performed on neonatal organotypic cochlear cultures and adult acutely isolated cochlear explants. In adult explants, bath application of ATP resulted in a dose-dependent generalized immediate increase in [Ca^2+^]_i_ in OHCs, IHCs, and pillar cells, which was significantly attenuated in the presence of 40 U/l apyrase, but not 100 μM CBX (Fig 4).

**Fig 5:**
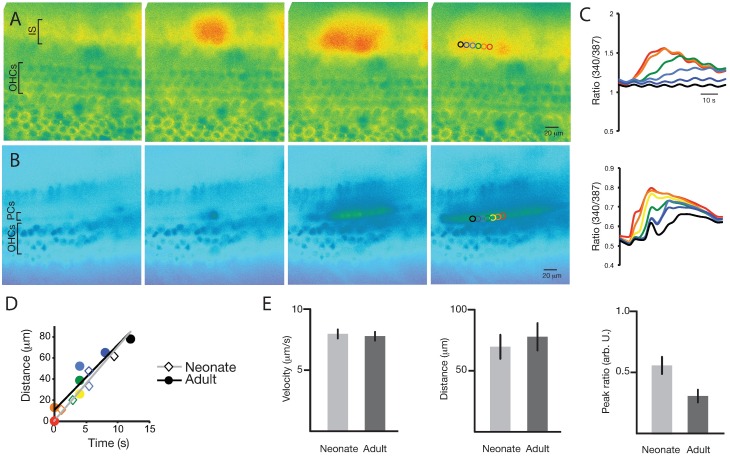
ICS wave activity in neonatal and adult cochleae. A. Neonatal culture ICS activity. A spontaneously occurring ICS wave in the inner sulcus (IS) region of a P3 neonatal cochlear culture was regularly seen to initiate within the IS and propagate bidirectionally along the longitudinal axis of the cochlea. Outer hair cell (OHC) rows are shown as reference. B. Adult explant ICS activity. A qualitatively similar ICS wave was seen to initiate and propagate within the pillar-cell (PC) row of the adult cochlear explant in response to acoustic overstimulation. C. ICS wave measurement. Graph of individual-cell [Ca^2+^]_i_ (colored circles) shows a peak propagating in time and space for both neonatal (upper) and adult (lower) cochlear explants. D. ICS wave velocity. Neonatal (light gray line, diamonds) and adult (black line, circles) wave velocities were calculated from the peaks in (C). E. ICS wave properties. Neonatal (light gray) and adult (dark gray) ICS waves exhibited similar velocity (left) and propagation distance (center). Neonatal waves had somewhat increased ICS wave amplitude (right), but this was highly variable. N = 7 waves, each from a unique, independent culture or explant, were examined for each. Bars indicate means; lines indicate standard errors of the mean.

In the neonatal cultures, increases in [Ca^2+^]_i_ were initiated in individual cells of the inner and outer sulcus both spontaneously and in response to exogenously applied 1 μM ATP (Fig 5A). This increase in [Ca^2+^]_i_ then propagated away bidirectionally from the original site along the longitudinal axis of the cochlea. In adult acutely isolated cochlear explants, spontaneous intracellular Ca^2+^ transients were not observed. Delivery of loud sound (103 dB, 1 kHz) or direct mechanical damage to an OHC with a 2-3-μm micropipette elicited ICS waves (Fig 5B) that propagated bidirectionally along the pillar cells. Closer investigation of sound-induced ICS events revealed that each was preceded by spontaneous death of an OHC.

ICS wave velocity was measured by identifying the peak [Ca^2+^]_i_ at positions along the axis of wave propagation for neonatal and adult cochleae (Fig 5C and 5D). Wave propagation velocity and distance were similar for adult and neonatal cochleae (7.8 (95% confidence interval (CI): 7.0–8.5) μm/s vs 8.2 (7.4–9.1) μm/s (*p* = 0.49) and 78.0 (56.7–99.3) μm vs 68.9 (51.8–86.0) μm (*p* = 0.53), respectively (N = 7 for each)). Peak fluorescence ratio (340/387) change, was significantly higher in the neonatal cultures (0.31 (0.20–0.42) vs 0.55 (0.42–0.68), respectively (*p* = 0.016); Fig 5E). No propagating waves were seen upon sound exposure, ATP exposure, direct pipette damage, or spontaneous OHC death in the presence of 100 μM CBX or 40 U/l apyrase (data not shown).

### Noise-induced hearing loss in gap-junction-deficient Cx26 conditional KO mice

The *in vitro* experiments in the adult gerbil cochlea described above suggested that acoustic overstimulation induces early changes in [Ca^2+^]_i_ as well stochastic triggering of ICS waves that are both dependent upon extracellular ATP and sensitive to blockade by CBX. Individuals with isolated disruption of ICS activity but intact junctional conductance owing to V84L Cx26 mutation have hearing loss. We hypothesized that Cx26 deficiency could thus predispose to noise-induced hearing loss owing to disruption of ICS activity. To investigate this, we evaluated noise-induced shifts in hearing thresholds in a TMX-inducible conditional KO model of Cx26-associated hearing loss, which avoids the developmental consequences of Cx26 dysfunction while permitting the evaluation of its functional effects [16].

**Fig 6:**
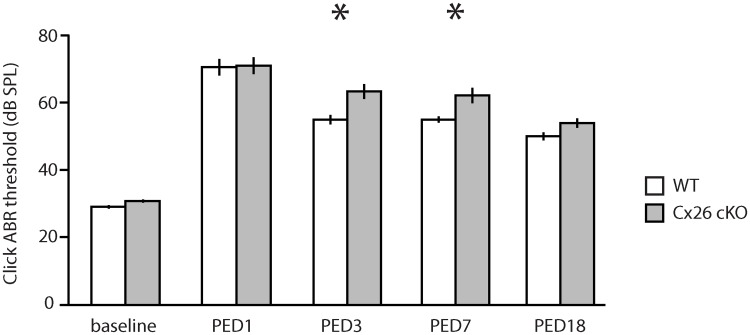
Noise-induced threshold shift in Cx26 cKO mice. Click ABR thresholds were measured in wild-type (WT, white, N = 7) and Cx26 conditional knockout (Cx26 cKO, gray, N = 8) mice before (baseline) and 1, 3, 7, and 18 days (PED1-18) after exposure to 1 hr of 8–16 kHz octave-band noise at 106 dB SPL. Bars indicate means; lines indicate standard errors of the mean; asterisks indicate *p* < 0.05 by unpaired t-test.

Administration of TMX to P8-10 Cx26 cKO mice gave rise to normal hearing at postnatal day 21 (P21), as previously described [17]. In wild-type (WT) mice (N = 7), 1-hr, 106 dB SPL, 8–16 kHz octave-band noise elicited a large temporary threshold shift (TTS) with modest recovery and significant permanent threshold shift (PTS) 18 days post-noise exposure (PNE). Cx26 cKO mice (N = 8) had identical hearing thresholds at baseline and one day after noise exposure (PNE1), but had slightly impaired recovery over the following 7 days (Fig 6), suggesting a mild impairment in the cochlear restorative response to acoustic trauma.

## Discussion

Ca^2+^ plays an important role in the cochlea. Whereas Ca^2+^ dynamics in the cochlea have been studied extensively in specialized compartments around the apical hair bundle and basolateral synapse of hair cells [5], the roles of Ca^2+^ in cellular homeostasis and regulation, especially in cochlear supporting cells, are poorly understood. In this study, we explored the role of intracellular Ca^2+^ in the cochlea's response to acoustic overstimulation by performing ratiometric Ca^2+^ in a physiologically viable *in vitro* explant preparation of the gerbil cochlea. We demonstrate that ATP-dependent changes in [Ca^2+^]_i_, as well as ICS waves very similar to those seen in neonatal cochlear cultures, can be elicited in the presence of physiologic levels of acoustic overstimulation. Acoustic overstimulation induces changes in [Ca^2+^]_i_ in all cell types; these changes are dependent upon extracellular ATP, connexin hemichannel release of ATP, and mechanoelectrical transduction, and occur within tens of seconds of sound exposure. Acoustic overstimulation or direct mechanical trauma also produces ATP-dependent, CBX-sensitive sporadic ICS waves in pillar cells that are very similar to those seen and previously characterized extensively in supporting cells in neonatal cochlear cultures. These findings provide proof of principle that the phenomenon of trauma-induced, ATP- and connexin-dependent ICS waves persists in the hearing cochlea with physiologic stimuli.

Compared to Ca^2+^ waves seen in neonatal cultures, adult cochlear ICS waves were markedly less frequent and showed significantly less change in absolute [Ca^2+^]_i_; in fact, these waves were only infrequently captured even in the presence of acoustic overstimulation. This may be due to a greatly increased threshold for ATP-induced [Ca^2+^]_I_ increase- though we were able to elicit increases in [Ca^2+^]_I_ with exogenous ATP, high concentrations of ATP were required, in the tens of micromolar. This is in contrast to the [Ca^2+^] increases in tens of nanomolar measured in endolymph after sound overexposure *in vivo* [2]. The local concentration of ATP at the reticular lamina, however, in the context of paracrine signaling during regenerative Ca^2+^ waves may be much higher, and has been modeled as high as 2 mM [3]. A change in the threshold of ATP induction of Ca^2+^ influx might change the kinetics of Ca^2+^ wave propagation—we found that the magnitude of wave-associated [Ca^2+^]_I_ changes was lower in the adult cochleae.

On the other hand, other ntrinsic characteristics of the adult cochlear ICS waves—in particular, their qualitative behavior, propagation distance, and velocity—were identical to their neonatal counterparts, suggestive of a largely similar fundamental mechanism. Previous studies have shown that these waves, which propagate only through supporting-cell networks, are dependent upon apical connexin-based hemichannels, exogenous ATP signaling through purinergic receptors, transmission of Ca^2+^ second messengers including IP_3_ through gap junctions between supporting cells, and release of Ca^2+^ from intracellular endoplasmic reticular stores [3–7]. It was previously found that spontaneous ICS activity ceases by P16 in Kölliker's organ, a transient gap-junction-interconnected structure in the inner sulcus of the developing cochlea [20]. Here, we corroborate the absence of spontaneous ICS activity, but demonstrate an ability to induce ICS waves with physiologic sound stimuli, suggesting that this quiescent developmental phenomenon can become reactivated upon traumatic insult. Better understanding of ICS waves in the adult cochlea may be an opportunity to re-activate other more immature developmental pathways in the cochlea, including hair-cell regeneration.

This study demonstrates the existence of two separate responses of intracellular Ca^2+^ to acoustic overstimulation—monotonic accumulation of Ca^2+^ and discrete ICS waves. This accumulation of [Ca^2+^] in cochlear cells upon acoustic overstimulation is largely consistent with prior observations of [Ca^2+^]_i_ changes in an isolated guinea pig temporal bone preparation [28]. Sound overstimulation is well known to elicit ATP production in endolymph [2], likely through release from connexin hemichannels [6], which acts upon P2X_2_ receptors, which are expressed in a broad set of cochlear cell types and have been implicated in protection against noise-induced hearing loss [29,30]. Purinergic signaling leads to release of Ca^2+^ from intracellular stores [6,7]. Accumulation of intracellular Ca^2+^ is subsequently well known to be a precursor to apoptosis in many cell types in the cochlea [31]. Our pharmacologic manipulations, which implicate ATP, hemichannels/gap junctions, mechanoelectrical transduction, and Ca^2+^ reuptake, are consistent with this set of mechanisms. The Ca^2+^ changes seen in our study are small, and though an increase in [Ca^2+^] is consistently seen early during sound stimulation in IHCs and pillar cells, findings in OHCs, and at later timepoints, are more variable. This is in contrast to prior findings of consistent OHC Ca^2+^ accumulation upon sound overstimulation [28] and may reflect significant differences in explant preparations. Evaluation of sound-induced Ca^2+^ changes in a truly *in vivo* model would be necessary to resolve these differences.

The role of the ICS waves, on the other hand, is less clear. On one hand, when ICS waves are activated after cochlear trauma, hair-cell death ensues through ERK1/2 signaling in supporting cells [8]. On the other hand, ICS activity is abolished by gap-junction inhibition or Connexin 26 knockout [6], and the V84L mutation in Connexin 26, which retains junctional conductance but has diminished IP_3_ permeability, is sufficient to cause hearing loss in humans, suggesting that ICS waves are protective [18]. We found that inducible conditional Cx26 knockout mice, which develop hearing normally at P21, have similar initial threshold shifts to WT mice 1 day after noise exposure, but modest, though statistically significant, impaired recovery towards baseline. This finding suggests that ICS signaling waves triggered upon initial acoustic overstimulation may indeed be protective, and that disruption of these waves in the Cx26 knockout may impair the animal's ability to recover from temporary hearing loss.

The organ of Corti functions in a highly regulated ionic and micromechanical environment that cannot be perfectly replicated, even with this two-chamber explant system; therefore, this study remains limited. ICS waves were not consistently observed, and late changes in [Ca^2+^]_i_ on the order of minutes after initiation of sound were highly variable. This experimental variability may be an artifact of the explant system, or may represent true physiologic variability. Evaluation of these homeostatic Ca^2+^ transients in the adult cochlea *in vivo* would be necessary to further explore their effect on downstream cell regulatory pathways.

Nevertheless, this demonstration of sound-induced homeostatic Ca^2+^ signaling in the cochlea as an early response to acoustic overstimulation is reminiscent of ICS waves in the glia and retina, which have been implicated in a wide array of sometimes contradictory downstream cell homeostatic and regulatory functions, including neuronal guidance, synaptic maintenance, apoptosis, and cellular regeneration [13]. The language of ICS activity, and how it drives different potential outcomes, however, is not known. Identification of this pathway in the adult, hearing cochlea may provide a new target for future interventions for both noise-induced and Connexin 26-associated hearing loss.
